# A workflow for expanding DNA barcode reference libraries through ‘museum harvesting’ of natural history collections

**DOI:** 10.3897/BDJ.11.e100677

**Published:** 2023-05-10

**Authors:** Valerie Levesque-Beaudin, Meredith E. Miller, Torsten Dikow, Scott E. Miller, Sean W.J. Prosser, Evgeny V. Zakharov, Jaclyn T.A. McKeown, Jayme E. Sones, Niamh E Redmond, Jonathan A. Coddington, Bernardo F. Santos, Jessica Bird, Jeremy R. deWaard

**Affiliations:** 1 Centre for Biodiversity Genomics, University of Guelph, Guelph, Canada Centre for Biodiversity Genomics, University of Guelph Guelph Canada; 2 National Museum of Natural History, Smithsonian Institution, Washington, DC, United States of America National Museum of Natural History, Smithsonian Institution Washington, DC United States of America; 3 Department of Integrative Biology, University of Guelph, Guelph, Canada Department of Integrative Biology, University of Guelph Guelph Canada; 4 School of Environmental Sciences, University of Guelph, Guelph, Canada School of Environmental Sciences, University of Guelph Guelph Canada

**Keywords:** DNA barcoding, Diptera, museum harvesting, COI, arthropods, digitisation, National Museum of Natural History, USNM, Centre for Biodiversity Genomics

## Abstract

Natural history collections are the physical repositories of our knowledge on species, the entities of biodiversity. Making this knowledge accessible to society – through, for example, digitisation or the construction of a validated, global DNA barcode library – is of crucial importance. To this end, we developed and streamlined a workflow for ‘museum harvesting’ of authoritatively identified Diptera specimens from the Smithsonian Institution’s National Museum of Natural History. Our detailed workflow includes both on-site and off-site processing through specimen selection, labelling, imaging, tissue sampling, databasing and DNA barcoding. This approach was tested by harvesting and DNA barcoding 941 voucher specimens, representing 32 families, 819 genera and 695 identified species collected from 100 countries. We recovered 867 sequences (> 0 base pairs) with a sequencing success of 88.8% (727 of 819 sequenced genera gained a barcode > 300 base pairs). While Sanger-based methods were more effective for recently-collected specimens, the methods employing next-generation sequencing recovered barcodes for specimens over a century old. The utility of the newly-generated reference barcodes is demonstrated by the subsequent taxonomic assignment of nearly 5000 specimen records in the Barcode of Life Data Systems.

## Introduction

Digitally capturing biological data is an ongoing challenge, as classification, description, digitisation and collation of data can be tedious and time-consuming processes (Fontaine et al. 2012). Natural history collections (NHCs) are critically important biorepositories for billions of preserved biological voucher specimens and data and provide an extensive and fundamental record of the earth’s biodiversity (Lane 1996, Graham et al. 2004, Suarez and Tsutsui 2004, Ward 2012, Holmes et al. 2016, Yeates et al. 2016). Not only do NHCs contain representatives of the vast majority of the world’s described taxa, they also hold large proportions of currently undescribed species (Hebert et al. 2013). Developing efficient workflows for recovering DNA and biological data from NHCs is critical to making this information available for emergent projects in biodiversity and to help build reliable DNA reference libraries. Digitised specimen information, voucher images, genomic samples and molecular data are increasingly being captured from NHC specimens and stored in online repositories, such as the Barcode of Life Data Systems (BOLD; Ratnasingham and Hebert 2007), the Global Biodiversity Information Facility (GBIF; see Edwards (2004)), the Global Genome Biodiversity Network (GGBN; Droege et al. 2014) and GenBank (Sayers et al. 2021). Unfortunately, the addition of NHC data and resources to these repositories have remained relatively low and would benefit greatly from refined workflows that could increase the scale and uptake of this invaluable data source.

‘Museum harvesting’ refers to the selection, digitisation and sampling of identified voucher specimens held in NHCs, for the purpose of isolating and sequencing one or more barcode markers – a short fragment of the cytochrome c oxidase I (COI) gene in the case of animals (see Hebert et al. (2013)). The COI barcode region has been demonstrated to reliably delineate species in a wide range of taxa, most often in concordance with Linnaean taxonomy (Hausmann et al. 2013, Lopez-Vaamonde et al. 2021) and a persistent registry of these molecular operational taxonomic units, called Barcode Index Numbers (BINs; Ratnasingham and Hebert 2013) is maintained on BOLD. In the case of arthropod taxa, the focus of the present study, museum harvesting typically involves the subsampling of a leg from a pinned or ethanol-preserved museum voucher. For minute specimens, the entire specimen can be used for non-destructive lysis and DNA extraction, with an added step of recovering the voucher (Porco et al. 2010). With the use of a routine and efficient workflow, museum harvesting can be an optimal strategy to build or add to a validated barcode reference library and to capture the valuable data they hold.

The Smithsonian Institution’s National Museum of Natural History (NMNH, USNM; https://naturalhistory.si.edu/) in Washington, D.C., maintains one of the largest arthropod collections in the world, holding over 35 million insect specimens alone (Smithsonian Institution 2021). Over the last decade, the Centre for Biodiversity Genomics (CBG; https://biodiversitygenomics.net/) at the University of Guelph has partnered with the NMNH to develop streamlined and effective museum harvesting methods. To date, over 120,000 specimens have been DNA barcoded and digitised through this partnership. Early museum harvesting efforts were hampered by DNA degradation due to specimen age and preservation method (Hebert et al. 2013), but recent advancements in high-throughput sequencing-based approaches (e.g. Prosser et al. (2016), Hebert et al. (2018)) have significantly improved the recovery of DNA barcodes from older, rare or poorly-preserved specimens. The cost- and time-efficient methods for museum harvesting advanced through this partnership, paired with improved barcode analysis methods, are allowing for the assembly of barcode libraries using museum specimens (Hebert et al. 2013) and the archiving of valuable DNA derivatives (Coddington et al. 2016).

This present study focused on the museum harvesting of true fly (Diptera) specimens held at the USNM, a collection that comprises over 3,200,000 pinned specimens and over 55,000 identified species from 162 families (Smithsonian Institution 2021). By targeting 33 families with previously limited coverage in the BOLD reference library (https://www.boldsystems.org/index.php/Taxbrowser_Taxonpage?taxon=Diptera&searchTax=Search+Taxonomy), the objectives were to further develop museum harvesting workflows, to barcode authoritatively identified specimens and to explore the ability of the generated barcodes to guide the classification of unidentified BINs on BOLD. A detailed workflow for museum harvesting of identified voucher specimens is described, outlining methods for both on-site specimen processing at a NHC (such as the USNM) and off-site processing at a laboratory facility (such as the CBG). The process for releasing data and valuable derivatives is also explained in detail and demonstrated with the 941 dipteran specimens analysed herein, with data, images and bioresources available through multiple platforms including BOLD, GBIF, GGBN, GenBank and the public USNM collections database.

## Material and methods

**Figure 1.**
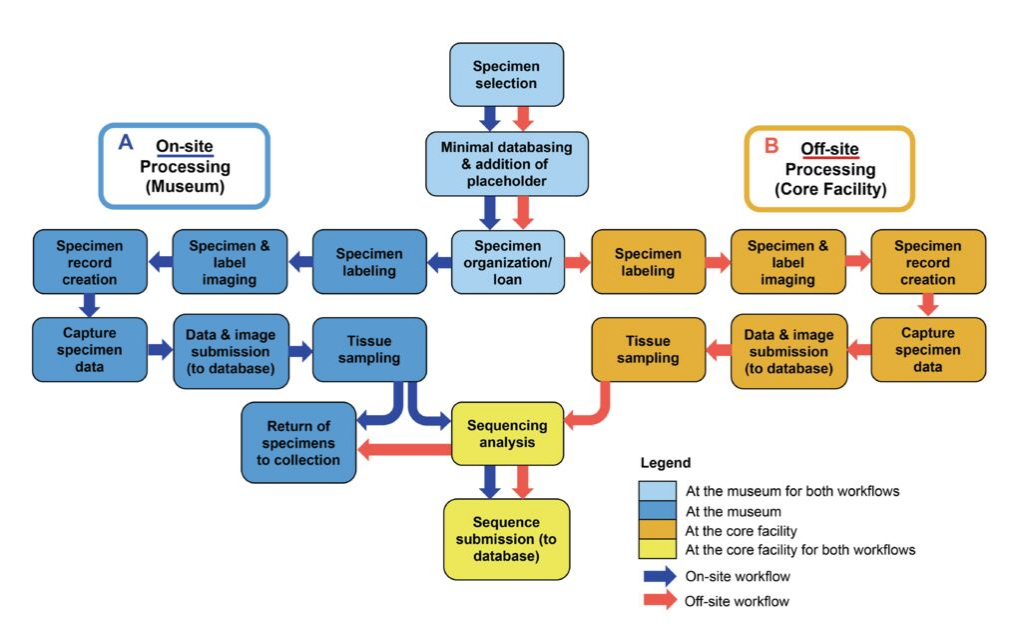
Generalised workflow for ‘museum harvesting’, for both A) on-site and B) off-site specimen processing.

Museum harvesting can be completed through on-site and off-site processing workflows (Fig. 1). On-site museum harvesting involves the majority of specimen processing being physically completed at the museum, herbarium or other NHC. This on-site work includes specimen selection, labelling, imaging, databasing, data record creation/submission, tissue sampling and voucher specimen return, which are all completed prior to barcode analysis (Fig. 1A). After on-site processing, tissue samples are transported to the off-site (laboratory) facility for barcode analysis and submission of sequences to the associated sequencing databases. For off-site museum harvesting, after specimen selection and specimen loan preparation are completed on-site, all subsequent steps of labelling, imaging, databasing, data record creation/submission, tissue sampling, barcode analysis and submission of sequences to the associated sequencing databases are completed at the off-site facility (Fig. 1B). After sequencing is complete, specimens are returned to the on-site facility. In this study, museum harvesting was completed using both on-site and off-site workflows and is described in detail in the following subsections.

### Specimen selection at USNM

Staff from CBG completed two visits to the USNM, Department of Entomology in 2017 (2-6 October 2017 and 4-12 December 2017). Prior to the first research visit, Orthorrhapha (Diptera) was selected as a target taxonomic group. CBG staff prepared a list of Orthorrhapha genera and species lacking representation in BOLD to assist with on-site specimen selection.

**Figure 2.**
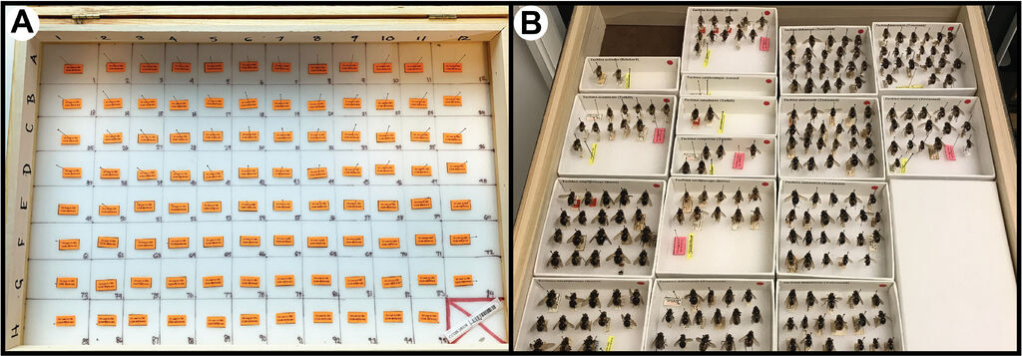
Examples of A) a Schmitt box with placeholder labels and B) the placeholder labels used in a specimen drawer at USNM.

To streamline subsequent processing of specimens, museum harvesting was completed using Schmitt insect boxes arrayed with 8 x 12 grid squares matching a 96-well microplate layout used in the sequencing laboratory (columns numbered from 1 to 12 and rows labelled from A to H). Each Schmitt box accommodates 95 pinned specimens, with the 96th square reserved for a negative control. Ten Schmitt boxes were assigned a unique alphanumeric barcode label received from the Canadian Centre for DNA Barcoding (CCDB; http://ccdb.ca/; e.g. CCDB-31120). The same unique alphanumeric barcode was used to create a unique sample ID for each of the 95 squares of the array (e.g. CCDB-31120-A01). Placeholder labels (“removal labels”) for all sample IDs were pinned in each square matching the corresponding sample ID (Fig. 2A). These removal labels were used as a placeholder to temporarily replace the corresponding voucher within unit trays/drawers (Fig. 2B) in the Diptera collection during specimen selection at the USNM collection and to permit quick and accurate return of the specimens once the loan had been completed (see below).

**Figure 3.**
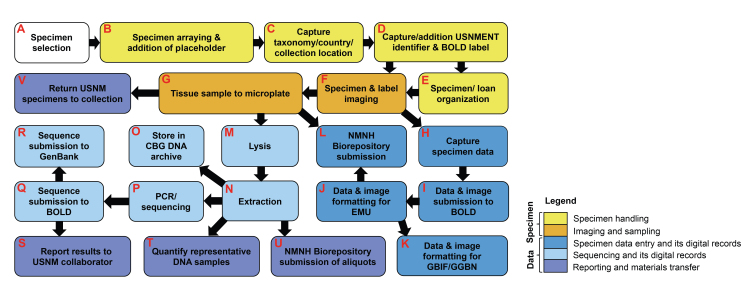
Workflow for ‘museum harvesting’ at USNM.

Specimens were selected in the museum by moving systematically through each adjacent row, cabinet and insect drawer of the target families within the insect collection to search for genera on the target list (Fig. 3A). At least one voucher specimen, representing each target genus was selected, with two or more distinct species selected whenever possible. Factors that were considered when selecting specimens from the collection included specimen age, collection method (if available on label), specimen condition, associated data (e.g. record of rearing or dissection), any specific curator instructions, as well as the number of specimens and/or species present for each target genus.

For each specimen selected and removed from its cabinet/drawer location and placed into a square in a Schmitt box array, the corresponding removal label was placed into the unit tray within the cabinet/drawer and replaced when the specimen was returned to the USNM (Fig. 3B). Taxonomy, country of collection, sample ID and specimen cabinet/drawer locations for each specimen were carefully recorded by CBG staff (Fig. 3C). During the two research visits, ten arrays of 95 Diptera specimens each (950 specimens total) were selected for processing. Off-site harvesting was completed at CBG for four arrays, which were selected during the first research visit (CCDB-31122 to CCDB-31125). On-site museum harvesting was completed at USNM for the remaining six arrays selected during the second research visit (CCDB-31120, CCDB-31121, CCDB-31126 to CCDB-31129).

### Specimen processing at CBG

During the first research visit in October 2017, after specimen selection was completed for the first four arrays, a report of the taxonomy, country of collection, sample ID and specimen cabinet/drawer locations was provided to the USNM curator (T.D.) for use in preparing the specimen loan (Fig. 3E). After the loan was approved by the collections manager and documentation was sent to the US Fish and Wildlife Service, the four specimen arrays were transferred to CBG. Once transferred to CBG, specimen labels were added by CBG staff. These labels included unique sample IDs and BOLD process IDs, as well as scannable USNMENT unique specimen identifiers (unless a USNMENT label was already present) (Fig. 3D).

Multiple habitus photos of each specimen were taken in the CBG imaging lab using a Canon EOS 70D camera (Fig. 3F) and stacked into one image using Helicon Focus (Helicon Soft. Ltd.; https://www.heliconsoft.com/). Labels from each specimen were removed and imaged, and then carefully placed back on to the specimen in the original order. Digitisation of specimen label data was completed using the label images, entered into the BOLD submissions spreadsheet and submitted to BOLD (into the ASILO project) (Fig. 3H). Tissue sampling was completed by removing two legs (a middle leg and a hind leg) from the same side from each specimen, placing one into an assigned microplate for each array and the second into a tissue archiving plate (Fig. 3G). This sampling of a second leg for tissue archiving is optional and is typically skipped for most projects at the CBG or USNM. Sampling equipment was sterilised using alcohol and flame between tissue samples of each individual specimen, following the CCDB protocol (Ivanova et al. 2007) and all applicable safety procedures. All necessary precautions were taken to prevent cross-contamination of and/or damage to the specimens during imaging and tissue sampling. Microplates were submitted to CCDB for sequencing (Fig. 3M-R) and the tissue archiving plates were given to USNM staff to be deposited in the NMNH Biorepository (https://naturalhistory.si.edu/research/biorepository) (Fig. 3L). This process was repeated for all selected specimens in all four arrays. Once processing was complete, specimens were returned to the USNM during the second research visit in December 2017 (Fig. 3V). Upon return to the USNM, after going through the pest management freezer cycle, specimens were returned to their original locations in the collection using a prepared list of cabinet locations and the removal labels associated with each specimen.

### Specimen processing at the USNM

During the second research visit in December 2017, after specimen selection for the remaining six arrays was completed (Fig. 3A-C) and approved by museum curators, specimen labels and scannable USNMENT unique specimen identifiers were added to each specimen (unless a USNMENT label was already present) (Fig. 3D). To adhere to time constraints, a single habitus image of the corresponding specimen (with labels removed) was taken with the same camera using a tripod mount on a white portable background (Fig. 3F). Labels from each specimen were imaged and then carefully placed back on to the specimen in the original order. This was repeated for all selected specimens in all six arrays.

Tissue sampling was completed by removing two legs (a middle leg and a hind leg) from each specimen, placing one into an assigned microplate for each array and the second into a tissue archiving plate (Fig. 3G). Since this was executed off-site, sampling equipment was instead sterilised using an ELIMINase bath, followed by three baths of distilled water between tissue samples of each individual specimen (see Centre for Biodiversity Genomics (2021)). While sterlisation by alcohol and flame may be preferable for sampling museum specimens, access and permission to use flame or hazardous reagents may be restricted at off-site NHCs, so the use of a decontamination detergent (e.g. ELIMINase) or 10% bleach solution is a comparable alternative. All necessary precautions were taken to prevent cross-contamination of and/or damage to the specimens during imaging and tissue sampling. Microplates were brought back to CBG and submitted to CCDB for sequencing (Fig. 3M-R) and the tissue archiving plates were given to USNM staff to be deposited in the NMNH Biorepository (Fig. 3L). This process was repeated for all selected specimens in all six arrays.

Once tissue sampling was completed at USNM, specimens were returned to their original locations in the collection using a prepared list of cabinet locations and the removal labels associated with each specimen. Databasing of label data was completed using the label images and entered into the BOLD submissions spreadsheet and submitted to BOLD (in the ASILO project) (Fig. 3H).

### Laboratory analysis

The 941 tissue samples were lysed and extracted following the silica-based protocol outlined in Ivanova et al. (2006) (Fig. 3M-N). A volume of 50 μl of lysis buffer (30 mM Tris-HCl at pH 8.0, 700 mM guanidine thiocyanate, 30 mM EDTA with pH 8.0, 0.5% Triton X-100, 5% Tween-20 and 2 mg/ml proteinase K) was added to each well and incubated at 56°C for 18 h. Following incubation, 100 μl of Binding Mix (5 mM Tris-HCl at pH 6.4, 3 M guanidine thiocyanate, 10 mM EDTA with pH 8.0, 2% Triton X-100 and 50% ethanol) was added to each lysate and the 150 μl was transferred to a silica membrane plate (PALL Corporation). The membrane was washed with 180 μl of Protein Wash Buffer (2.6 mM Tris-HCl at pH 6.4, 1.56 M guanidine thiocyanate, 5.2 mM EDTA with pH 8.0, 1.04% Triton X-100 and 70% ethanol) and 700 μl of Wash Buffer (10 mM Tris-HCl at pH 7.4, 50 mM NaCl, 0.5 mM EDTA with pH 8.0 and 60% ethanol). The membrane was then dried and DNA was eluted into a new 96-well microplate with 40 μl of Elution Buffer (10 mM Tris-HCl at pH 8). Following the completion of all laboratory steps, the genomic DNA extracts were split (20 μl each) with one half stored in the CBG DNA archive (Fig. 3O) and the other sent to the NMNH Biorepository (Fig. 3U).

PCR amplification and sequencing was first completed using Sanger sequencing and analysis (Fig. 3P) following Hebert et al. (2013). This process used two primer cocktail sets, (C_LepFolF+MLepR2 and MLepF1+C_LepFolR), targeting overlapping fragments of the COI gene, 307 and 407 base pairs (bp) in length, respectively (see Hebert et al. (2013) for primer sequences and references).

Each PCR reaction consisted of 2 µl of DNA template added to the appropriate well in pre-made, 96-well PCR plates, with 6.25 µl of 10% D-(+)-trehalose dihydrate (ThermoFisher Scientific), 2 µl of Hyclone ultra-pure water (ThermoFisher Scientific), 1.25 µl of 10× PlatinumTaq buffer (Invitrogen by ThermoFisher Scientific), 0.625 µl of 50 mM MgCl_2_ (Invitrogen by ThermoFisher Scientific), 0.125 µl of each primer, 0.0625 µl of 10 mM dNTP (KAPA Biosystems) and 0.060 µl of 5 U/µl PlatinumTaq DNA Polymerase (Invitrogen by ThermoFisher Scientific) for a total reaction volume of 12.5 µl. Thermal cycling conditions were 94°C for 1 min, 5 cycles at 94°C for 40 s, 45°C for 40 s, 72°C for 1 min, followed by 35 cycles at 94°C for 40 s, 51°C for 40 s, 72°C for 1 min and a final extension at 72°C for 5 min. All amplicons were visualised on a 2% agarose E-gel 96 pre-cast gel (ThermoFisher Scientific). Following consolidation into 384-well plates, PCR clean-up was completed with CleanSeq bead-based purification (Agencourt Biosciences) and cycle sequencing was performed using a modified BigDye 3.1 Terminator (Applied Biosystems, ThermoFisher Scientific) protocol (Hajibabaei et al. 2005). Cycle sequencing conditions were 96°C for 1 min followed by 35 cycles at 96°C for 10 s, 55°C for 5 s, 60°C for 2.5 min and a final extension at 60°C for 5 min. Bi-directional sequencing was performed on an ABI 3730xl DNA Analyzer (Applied Biosystems, ThermoFisher Scientific), while traces were assembled and edited using CodonCode Aligner v. 10.0.2 (CodonCode Corporation). All sequences and trace files were uploaded to BOLD in the ASILO project (https://www.boldsystems.org/index.php/MAS_Management_DataConsole?codes=ASILO) (Fig. 3Q).

All specimens that failed to gain a sequence (N = 418) were selected for next-generation sequencing (NGS), based failure-tracking utilising the method of Prosser et al. (2016), modified for use on the Sequel platform (see Quicke et al. (2020), D'Ercole et al. (2021)). This nested, multiplex PCR approach was used to generate multiple, short, overlapping fragments spanning the entire COI barcode region for 95 specimens simultaneously. Each amplicon was labelled with sample-specific unique molecular identifiers (UMIs) and pooled for single molecule real time (SMRT) sequencing on the Sequel platform (PacBio; https://www.pacb.com/technology/hifi-sequencing/sequel-system/) (D'Ercole et al. 2021).

PCR amplification involved three rounds (in 96-well plates unless otherwise stated): PCR1 to produce a spectrum of COI amplicons from each DNA extract; PCR2 to generate short, overlapping amplicons flanked by PacBio “PB1” adapters; and PCR3 to add UMIs to the amplicons from each specimen so multiple samples could be pooled for sequencing. The first round of PCR consisted of two reactions per sample (PCR1.1, PCR1.2), with each reaction containing three forward primers spanning the barcode region and 5–6 reverse primers (all untagged primers; see Fig 1A in Prosser et al. (2016)). The PCR regime consisted of 94°C for 2 min, 60 cycles of 94°C for 40 s, 48°C for 40 s and 72°C for 30 s and a final extension of 72°C for 5 min. The amplicons generated by PCR1 (up to 12 possible amplicons per sample) were pooled and size selected (> 100 bp) via carboxylate-coated magnetic beads (SpeedBeads; Sigma Aldrich) by mixing the 12.5 μl PCR reaction with 14.4 μl of magnetic beads and incubating for 10 min at room temperature. The beads were then immobilised on a magnet and washed three times with 120 μl of 80% ethanol. The washed beads were dried before the amplicons were eluted with 30 μl of water for use in PCR2.

PCR2 consisted of six reactions (PCR2.1, PCR2.2, PCR2.3, PCR2.4, PCR2.5, PCR2.6) with three (PCR2.1, PCR2.3, PCR2.5) using PCR1.1 as template, while the others (PCR2.2, PCR2.4, PCR2.6) used PCR1.2. PCR2 reaction cocktails were the same as those employed for PCR1, except the primers were tailed with PB1 adapters, providing universal primer binding sites for subsequent fusion of the UMIs. The PCR regime consisted of 94°C for 2 min, 40 cycles of 94°C for 40 s, 48°C for 40 s and 72°C for 30 s and a final extension of 72°C for 5 min. Following thermocycling, all six PCR2 reactions were pooled for each sample and a 12.5 μl aliquot of each pool was bead-purified as after PCR1. The purified products were then used for PCR3 which added sample-specific UMI tags to the amplicons recovered from each specimen. Asymmetrical dual-tagging was employed by using 96 different forward primers and 96 different reverse primers; the UMI-tagged fusion primers were complementary to the PB1 adapters of the PCR2 primers. The PCR regime consisted of 94°C for 2 min, 20 cycles of 94°C for 40 s, 64°C for 40 s and 72°C for 1 min, with a final extension of 72°C for 5 min. After thermocycling, the PCR3 amplicons were pooled for preparing libraries for SMRT sequencing.

Template preparation was performed following PacBio recommendations for SMRT sequencing. Purification involved adding 400 μl of the library to 480 μl of AMPure-PB beads (all subsequent purifications were carried out using the same 1.2 x beads-sample ratio). End-repair, SMRTbell adapter ligation, primer annealing and polymerase binding were all completed using PacBio instructions. The polymerase-bound products were loaded onto a SMRT cell (1M v.2) via diffusion loading without prior enrichment at a concentration of 18 pM. Sequencing run parameters were set using SMRTLink version 5.0 and sequencing was completed on a PacBio Sequel system. Default run settings were used with a few exceptions: insert size was set to 500, movie time to 480 min, immobilisation time to 120 min and pre-extension time to 20 min. Following sequencing, the raw data were analysed using the CCS algorithm under the SMRT Analysis module of SMRTLink. Default settings were used with the following exceptions: the maximum and minimum subread lengths were set to 500 bp and 100 bp, respectively.

The raw sequence data were used to generate circular consensus sequences (CCS) on SMRTLink v.7 using a minimum predicted accuracy of 99%. The short CCS reads (downloaded in FASTA format) were then assembled (de novo) into longer COI barcode sequences by custom bash and R scripts (made accessible by D'Ercole et al. (2021)): i) reads were filtered by a minimum QV of 20 and a minimum length of 100 bp; ii) reads passing the quality filter were associated with their source specimen (which were themselves morphologically identified to at least genus) via the UMIs and assigned order-level taxonomy by comparison to a BOLD reference library; iii) to remove non-target sequences, reads that did not match their expected order assignment were omitted from further analysis; iv) reads passing the taxonomy filter were then assigned to an amplicon via their loci-specific primers; v) since the relative position of each amplicon within the COI barcode region was known, the reads were correctly positioned relative to each other in an alignment-free and reference-free manner; vi) once the reads were correctly positioned, a consensus sequence was generated. If only non-overlapping fragments were recovered, the intervening region was filled with ambiguous (N) bases, so that the final consensus sequence was contiguous. The final assembled sequences were validated manually by Neighbour-Joining analysis and by querying the BOLD ID Engine (https://www.boldsystems.org/index.php/IDS_OpenIdEngine). Once the sequences were determined to be free of errors, they were uploaded to BOLD in the ASILO project (Fig. 3Q).

### Data analysis

To assess the impact of a museum harvesting-based reference library on the identification of BINs or records on BOLD, data from a large-scale collecting effort from CBG, the Global Malaise Program (GMP; http://www.globalmalaise.org; Perez et al. (2015)), was analysed (in January 2022) to verify how many records were gained or would have gained an identification. To be more inclusive, GMP is defined here as specimens from GMP projects or Malaise trap projects that could fall under the GMP campaign on BOLD (see deWaard et al. (2019)).

All sequences uploaded to BOLD that matched criteria outlined in Ratnasingham and Hebert (2013) from the GMP project and the USNM Diptera project were assigned to a new or existing BIN by the BOLD algorithm. When an unidentified BIN from a GMP specimen matched a taxonomically identified BIN assigned to a USNM Diptera record, the taxonomy of the GMP record was updated to match the known identification (i.e. BIN taxonomy match) (Ratnasingham and Hebert 2013). If a GMP BIN record did not match a taxonomically identified USNM Diptera BIN record, the BOLD ID Engine (Ratnasingham and Hebert 2007) located the closest sequence matches through the BLAST algorithm. A sequence divergence of less than 5% resulted in a genus level identification for the BIN and less than 2% divergence resulted in a match at the species level. In both methods, taxonomy was only applied to the GMP records according to the lowest level without conflict within a BIN or amongst the top matches in the BOLD ID Engine results. All taxonomic assignments were confirmed through morphological review.

## Data resources

All specimen data, which were formatted for the USNM EMu Collection Management System, as well as all specimen and label images, were provided to USNM staff for data submission and archiving (Fig. 3J). Specimen and sample data were also formatted and submitted to GBIF and GGBN (Droege et al. 2016; Fig. 3K). The 20 μl DNA aliquots were submitted to the NMNH Biorepository (Fig. 3U) and are publicly available on a loan basis for follow-up studies. All sequencing records from the ASILO project are available in the BOLD dataset DS-ASILO (https://dx.doi.org/10.5883/DS-ASILO), on GenBank (Fig. 3R) under the accessions MG967748-MG968255 and MN410974-MN411313 in the BioProject PRJNA437652 (www.ncbi.nlm.nih.gov/bioproject/437652), on GBIF in the ‘NMNH Extant Specimen Records (USNM, US)’ occurrence dataset (Orrell and Informatics Office 2021; https://doi.org/10.15468/hnhrg3) and through the GGBN data portal (Droege et al. 2014; https://www.ggbn.org/ggbn_portal/search/result?voucherCol=NMNH%2C+Washington) and the NMNH/USNM public collections data portal (https://collections.nmnh.si.edu/search/ento/).

## Results

A complete list of the 941 USNM Diptera specimens (including USNMENT catalogue numbers, collection date, country of origin, taxonomy, BOLD process ID, BIN, sequence length, GenBank accession number and NMNH Biorepository number) is provided in Suppl. material 1. The original target list covered 863 unique genera. Once the data were cleaned and updated to the most current taxonomy, the specimens represented 32 families, 819 genera and 695 identified species collected from 100 countries. Specimens analysed were collected between 1901 and 2017 and had a mean collection year of 1979 (or mean age of 38 years at the time of analysis). Of the 819 selected genera, 742 genera were represented by one specimen, 53 genera were represented by two specimens, 13 genera were represented by three specimens and 11 genera were represented by four to seven specimens.

**Figure 4.**
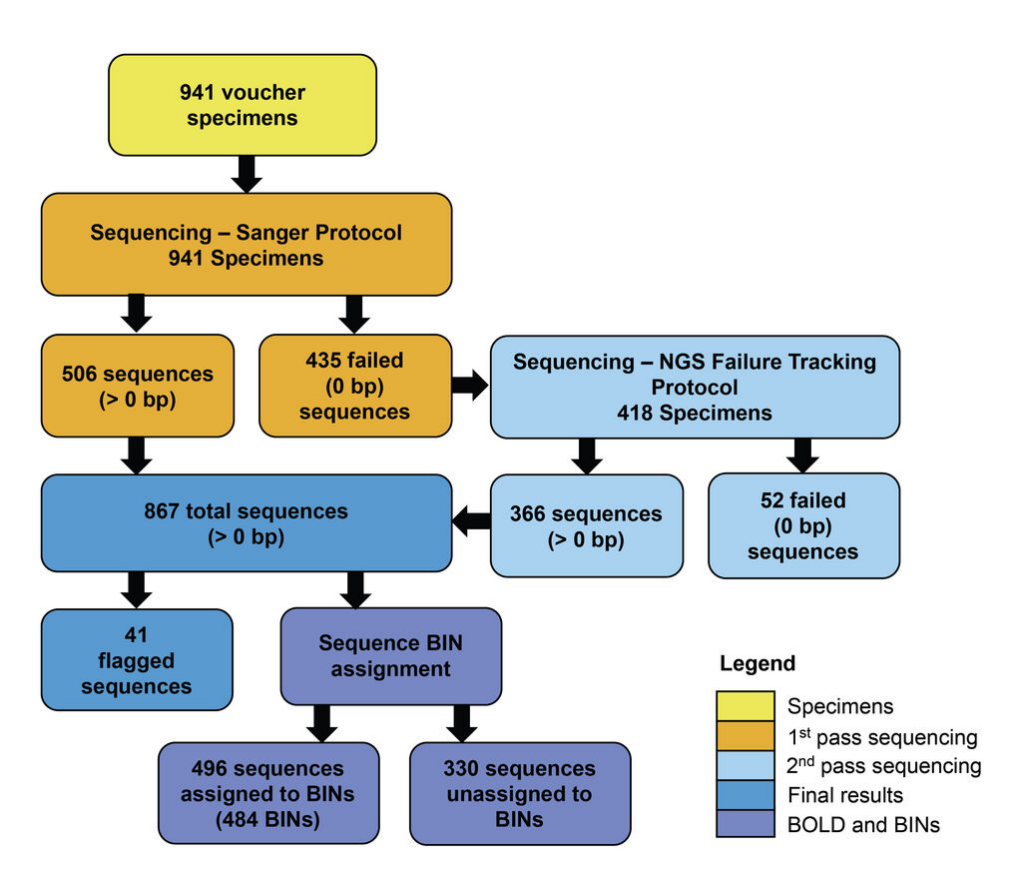
Breakdown of barcoding results for 941 USNM dipteran samples using Sanger-based and NGS-based failure-tracking protocols.

**Figure 5.**
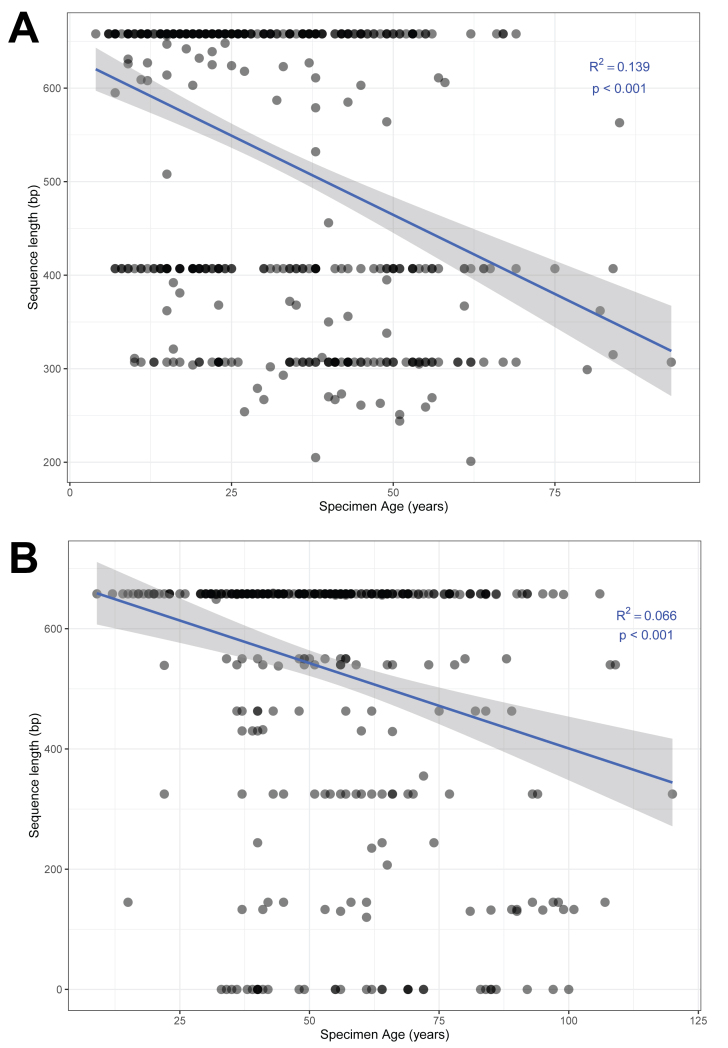
Analysis of the relationship between specimen age and sequence length for A) specimens sequenced using the Sanger-based protocol and B) specimens sequenced using the NGS-based failure-tracking protocol. Flagged records were excluded from these analyses. Note that all specimens that failed using the Sanger-based protocol were then attempted with the NGS-based failure-tracking protocol [and only appear in B)].

After sequencing using the Sanger-based method (Ivanova et al. 2006, Hebert et al. 2013), sequence recovery was 53.8% (506 of 941 specimens gained a barcode > 0 bp) (Fig. 4). Of the 506 specimens that gained a sequence, 489 sequences were barcodes of acceptable length (or 'acceptable barcodes', here defined as > 300 bp), resulting in an overall Sanger-based sequence success rate of 52.0% (mean sequence length = 527.6 bp, range = 201 bp to a full length of 658 bp). Of the 819 sequenced genera, 479 had acceptable barcodes recovered (56.8% success rate). The relationship between sequence length and the collection age of the specimen was significant (Fig. 5A, R^2^ = 0.139, p < 0.001) and unrelated to specimen taxonomy.

NGS-based failure-tracking was conducted on 418 specimens that did not gain a sequence during Sanger analysis. Of the 418 specimens, 366 gained a sequence (87.6%), bringing sequence recovery to 92.1% (867 of 941 total specimens > 0 bp). Of the 867 specimens, 824 had acceptable barcodes recovered (> 300 bp), resulting in an overall Sanger- and NGS-based sequence success rate of 87.6%. Of the 819 sequenced genera, 727 had acceptable barcodes recovered (88.8% success rate). For NGS-based failure-tracking, the relationship between sequence length and the collection age of the specimen was weaker, but still significant (Fig. 5B; R^2^ = 0.066, p < 0.001).

After NGS-based failure-tracking, of the 941 sequenced specimens, 41 records resulted in a contaminated barcode and were flagged on BOLD (17 at the time of sequencing using the Sanger-based protocol; 16 after the NGS-based protocol and eight flags were added after final data review) (Fig. 4). Of the 41 flagged records, 19 records were 400 bp or shorter (a sequence length often chosen to ensure overlap between the two amplicons). DNA barcodes gained from the Sanger and NGS sequencing methods were assigned to 484 BINs, 317 of which were new to BOLD (274 BINs were still unique on BOLD as of January 2022) (Fig. 4).

**Figure 6.**
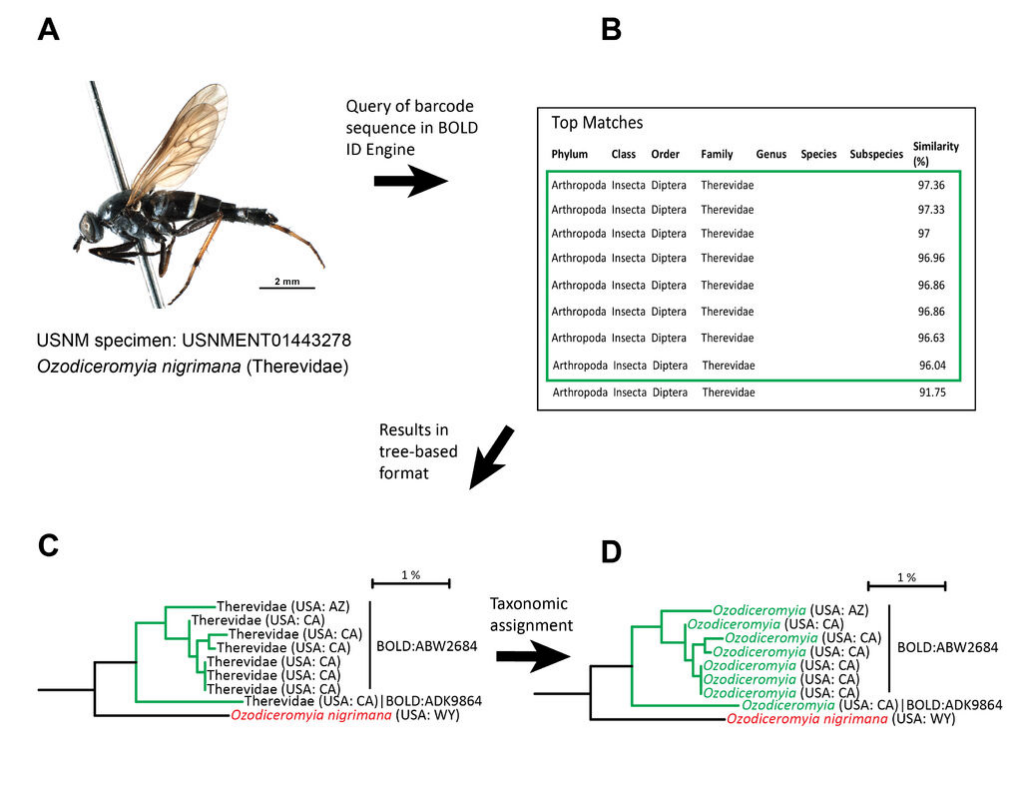
Taxonomic assignment of freshly-collected specimens through the addition of authoritatively identified USNM specimens to the BOLD reference library.

Using the taxonomically identified barcodes gained from the ASILO project that were greater than 400 bp, BOLD assigned (or could have assigned) genus- or species-level taxonomy to 4,999 specimens from the GMP project, through BIN taxonomy matches and BOLD ID Engine results (Fig. 6). Of the 4,999 specimens, the BIN taxonomy match assigned 1,263 specimens to the genus level and 2,403 specimens to the species level and the BOLD ID Engine assigned 1,333 specimens to the genus level and zero specimens to the species level (Table 1; note that no specimens or BINs could gain a species identification based on the BOLD ID Engine approach, as records with a BIN receive an identification first from the BIN taxonomy match approach).

## Discussion

Capturing biological data from natural history collections is critical for providing a comprehensive record of Earth’s biodiversity – both historical and contemporary. In our study, we aimed to develop and streamline a workflow for ‘museum harvesting’ of taxonomically identified voucher specimens held in NHCs. The workflow was then assessed through a pilot project that harvested and DNA barcoded 941 Diptera specimens archived in the Entomology collection of the Smithsonian National Museum of Natural History (USNM). Secondary objectives were to refine the museum workflow to be applicable to future projects at other NHCs and to demonstrate the utility of the newly-generated barcodes for the identification of previously unidentified specimens within the BOLD reference library. Utilising Sanger sequencing for initial DNA barcoding, followed by failure tracking using a NGS-based approach, 867 barcode sequences were recovered from the specimens with an overall sequencing success of 88.8% (727 of 819 sequenced genera gained a barcode > 300 bp).

Both on-site and off-site workflows were employed in the harvesting and barcoding of NHC specimens, each of which possesses advantages and poses challenges during various stages of voucher specimen processing. For on-site specimen processing, there is less risk of damage to fragile and often invaluable vouchers, as there is limited handling and no transport to an off-site location – only the tissue material for DNA extraction/sequencing must be moved off-site. The transport required for the off-site workflow poses a risk of specimen damage (and potentially specimen loss) and can be a time-consuming step if there is significant distance between both facilities, using either shipping or hand-carrying. On-site processing can also facilitate the harvesting of restricted specimens (e.g. from primary or secondary type series) that are not permitted to leave the collection, allow for taxonomic curators to work closely with technicians throughout the entire process and enable the voucher specimens to remain accessible as reference material. Conversely, the on-site workflow is significantly less cost- and time-effective, due to the longer time required within the NHC to complete the labelling, imaging, databasing and tissue sampling. This extra time adds supplemental costs, such as requiring additional technician hours to complete the work at the NHC and/or additional travel/accommodation expenses to compensate for the additional processing time. These tasks can be completed more efficiently at an off-site facility that has a dedicated team to accomplish each task and is better equipped to complete these steps in a shorter time period (e.g. optimised workspaces, superior imaging equipment, improved computational capacity for intensive processes, such as image stacking). In addition to more efficient completion of these crucial steps, off-site processing may also provide a more sterile environment for sampling, reducing the risk of contamination by exogenous DNA (Yeates et al. 2016).

The dipteran voucher specimens were DNA barcoded using two approaches: a Sanger-based method targeting two overlapping amplicons (Hebert et al. 2013) and a NGS-based method of failure tracking that targets six smaller fragments and employs the PacBio Sequel platform (Prosser et al. 2016, Quicke et al. 2020, D'Ercole et al. 2021). Sequence recovery (> 0 bp) and barcode compliance (> 300 bp) were both greatly improved after NGS-based failure tracking (53.8% to 92.1% and 52.0% to 88.8%, respectively). Although specimen age was a significant factor using the Sanger approach (R^2^ = 0.139, p > 0.001), its association was markedly weaker using the NGS-based approach, yet remained significant (R^2^ = 0.066, p > 0.001). The NGS-based approach has several advantages over the Sanger-based protocol, including increased success for much older voucher specimens (100+ years since collection) or specimens collected and preserved using methods that degrade DNA (D'Ercole et al. 2021). This increased success comes from targeting short fragments of COI, accommodating the fragmented DNA that is likely present in older and degraded samples. There are also some limitations of using the NGS-based approach, namely the higher sequencing costs compared to the Sanger-based approach, the increased processing time (for preparing multiple PCRs and more involved sequence editing/validation) and limited access to the proper infrastructure/equipment (e.g. liquid-handling robots, PacBio Sequel) required to complete the methods. The risk of contamination is also higher as it is more sensitive to amplifying trace amounts of DNA through the use of short amplicons and many PCR cycles. Although NGS-based approaches, including genome-skimming (e.g. Tin et al. (2014)), are likely the future of ‘museum harvesting’, Sanger-based methods can be a simple and effective approach, particularly for projects with budgetary constraints or for institutions and countries that lack infrastructure for NGS-based methods.

The DNA barcodes generated from the USNM voucher specimens were used to assign 4,999 records on BOLD to genus or species, through matching with an existing BIN or querying the new sequence through the BOLD ID Engine. This demonstrates the further utility of harvesting and barcoding authoritatively-identified museum specimens in the construction of reference barcode libraries: the addition of these records often enables more taxonomic assignments, expanding and refining the library further. These results reinforce the view that building reference libraries for many taxa can rely on a combination of museum harvesting (or other approaches where taxonomic assignments occur prior to barcode analysis) and the barcoding of freshly-collected, unidentified material that is assigned taxonomy after barcoding, through morphological assessment by an expert.

While this study was conducted on a small scale, with less than 1,000 voucher specimens, this workflow has formed the basis for larger-scale museum harvesting projects at the Smithsonian National Museum of Natural History and other institutions, using both on- and off-site processing. For example, Santos et al. (2022) describes subsequent efforts between the NMNH and the CBG to expand arthropod barcode reference libraries beyond Diptera. The study employed the off-site approach discussed here, but with modifications to permit the processing and analysis of a much wider taxonomic scope (13 orders and over 4500 genera) and to address the challenges certain taxa present (e.g. minute specimens of Hymenoptera < 2 mm in body size). It also represented a much larger scale of processing, covering over 8500 specimens across six separate specimen loans from the USNM. The study of Santos et al. (2022) demonstrates how the general workflow presented here should be broadly applicable and scaleable, and future projects will be able to customise this workflow, determining the ratio of on- and off-site processing to match their specific requirements and constraints. Much of this workflow should also be amenable to future developments in barcoding and sequencing approaches (e.g. genome-skimming; Bohmann et al. (2020)). Through museum harvesting workflows such as this, we can effectively and efficiently mine the rich biodiversity and genomic information stored in the world’s natural history collections and continue to build robust DNA reference libraries.

## Supplementary Material

726A8A2C-6360-5606-A36A-BC1D1A95B745 10.3897/BDJ.11.e100677.suppl1 Supplementary material 1 Summary data for the 941 USNM specimens selected for DNA barcoding Data type Specimen, sequence and voucher data Brief description Summary of specimen, sequence and voucher information for the 941 USNM specimens of Diptera analysed in the study. File: oo_652135.xlsx https://binary.pensoft.net/file/652135 Valerie Levesque-Beaudin, Meredith E. Miller, Torsten Dikow, Scott E. Miller, Sean W.J. Prosser, Evgeny V. Zakharov, Jaclyn T.A. McKeown, Jayme E. Sones, Niamh E. Redmond, Jonathan A. Coddington, Bernardo F. Santos, Jessica Bird and Jeremy R. deWaard

## Figures and Tables

**Table 1.** Global Malaise Program Records (GMP) that gained or could have gained taxonomy at the genus and species level using BIN taxonomy match and BOLD ID Engine approaches. These numbers are inclusive of older and newer Malaise trap projects that could fall under the large GMP campaign (see Materials and Methods for more details). *Covered by the BIN taxonomy match.

	**Gained genus assignment (records)**	**Gained species assignment (records)**	**Total**
**BIN taxonomy match**	1,263	2,403	**3,666**
**BOLD ID Engine**	1,333	*	**1,333**
**Total**	**2,596**	**2,403**	**4,999**
